# Effects of stem cell–derived exosome therapy on erectile dysfunction: a systematic review and meta-analysis of preclinical studies

**DOI:** 10.1093/sexmed/qfac019

**Published:** 2023-03-01

**Authors:** Yunpei Zhu, Tiancheng Jiang, Chi Yao, Jiawei Zhang, Chao Sun, Shuqiu Chen, Ming Chen

**Affiliations:** Department of Urology, Zhongda Hospital, Southeast University, Nanjing, 210009, China; Institute of Urology, Medical College, Southeast University, Nanjing, 210009, China; Department of Urology, Zhongda Hospital, Southeast University, Nanjing, 210009, China; Institute of Urology, Medical College, Southeast University, Nanjing, 210009, China; Department of Urology, Zhongda Hospital, Southeast University, Nanjing, 210009, China; Institute of Urology, Medical College, Southeast University, Nanjing, 210009, China; Department of Urology, Zhongda Hospital, Southeast University, Nanjing, 210009, China; Institute of Urology, Medical College, Southeast University, Nanjing, 210009, China; Department of Urology, Zhongda Hospital, Southeast University, Nanjing, 210009, China; Institute of Urology, Medical College, Southeast University, Nanjing, 210009, China; Department of Urology, Zhongda Hospital, Southeast University, Nanjing, 210009, China; Institute of Urology, Medical College, Southeast University, Nanjing, 210009, China; Department of Urology, Zhongda Hospital, Southeast University, Nanjing, 210009, China; Institute of Urology, Medical College, Southeast University, Nanjing, 210009, China; Zhongda Hospital Lishui Branch, Southeast University, Nanjing, 210009, China

**Keywords:** exosome, erectile dysfunction, intracavernous pressure/mean artery pressure, stem cell, structural changes, meta-analysis

## Abstract

**Introduction:**

Erectile dysfunction (ED) is a common disease among elderly men, and novel therapy methods are needed for drug-refractory ED. As an extracellular vesicle, stem cell–derived exosomes displayed erectile function improvement in rat ED models in some preclinical studies. However, the therapeutic efficacy has not been comprehensively evaluated.

**Aim:**

To study the therapeutic effects of stem cell–derived exosomes on ED in preclinical studies and to investigate the potential mechanisms responsible for the efficacy.

**Methods:**

The systematic literature search was conducted in Web of Science, PubMed, and Embase to retrieve studies utilizing stem cell–derived exosomes for ED treatment. We extracted data of intracavernous pressure/mean artery pressure (ICP/MAP), and cavernosum structural changes in rat ED models before and after stem cell-derived exosome therapy. RevMan 5.3 was used to perform meta-analyses of ICP/MAP and cavernosum microstructural changes. Publication bias was assessed with the Egger test and funnel plot by Stata 15.0 (StataCorp).

**Main Outcome Measures:**

Outcomes included ICP/MAP, smooth muscle, and endothelial markers—such as the ratio of smooth muscle to collagen and the expression of α-SMA (alpha smooth muscle actin), CD31 (cluster of differentiation 31), nNOS and eNOS (neuronal and endothelial nitric oxide synthase), TGF-β1 (transforming growth factor β1), and caspase 3 protein-to evaluate erectile function and microstructural changes. Forest plots of effect sizes were performed.

**Results:**

Of 146 studies retrieved, 11 studies were eligible. Pooled analysis showed that stem cell–derived exosomes ameliorated damaged ICP/MAP (standardized mean difference, 3.68; 95% CI, 2.64-4.72; *P* < .001) and structural changes, including the ratio of smooth muscle to collagen and the expression of α-SMA, CD31, nNOS, eNOS, TGF-β1, and caspase 3 protein. Subgroup analysis indicated that exosome type and ED model type made no difference to curative effects.

**Conclusion:**

This meta-analysis suggests the therapeutic efficacy of stem cell–derived exosomes for ED. Exosomes may restore erectile function by optimizing cavernosum microstructures.

## Introduction

Erectile dysfunction (ED) refers to the impotence to obtain or maintain an erection enough to permit satisfactory sexual intercourse.^1^ The incidence grows with age, especially in men aged >40 years, and it affects quality of life, causing physiologic and psychological problems.^2^ ED is an important complication in men with diabetes mellitus for its multifactorial pathophysiology, and more attention has been focused on postradical prostatectomy ED due to the growing incidence of prostate cancer in line with an increasing male life expectancy.^3^ Many other factors are reported to be involved with ED, such as cardiovascular diseases, metabolic syndrome, neuropathic damage, lower urinary tract symptoms, Peyronie disease (PD), obstructive sleep apnea, and psychiatric disorders.^4–7^ In animal models of ED, intracavernous pressure measurement for penile erection induced by electrical stimulation of the cavernous nerve has been widely adopted by researchers for evaluation of erectile function.^8^^,^^9^ It has been reported that ED was associated with decreased expression of endothelial markers (VEGF, endothelial nitric oxide synthase [eNOS], cluster of differentiation 31 [CD31], etc), smooth muscle markers (α-actin, smoothelin, etc), and pericyte markers (CD146 and NG2).^10^^,^^11^

In terms of therapies and management, oral phosphodiesterase type 5 inhibitors, such as sildenafil and tadalafil,^12^^,^^13^ were regarded as the first-line treatment of ED.^4^ Other treatment modalities include intracavernous injection therapy, testosterone therapy, vacuum constrictive devices, and penile prostheses. In addition, some researchers utilized low-intensity extracorporeal shock wave therapy and low-intensity pulsed ultrasound therapy to improve erectile function and penile hemodynamic by inducing neovascularization and promoting tissue regeneration.^14^^,^^15^ However, most of them are far from flawless. A certain proportion of patients with ED do not respond to phosphodiesterase type 5 inhibitors.^16^ Vacuum constrictive devices are expensive and may induce unnatural erections, which cannot meet the satisfaction of patients. Low-intensity extracorporeal shock wave therapy costs too much, and the actual physiologic changes of the penile tissue and the long-term risk of shock waves are not fully elucidated. Therefore, there is still a great need for more effective treatments that can provide long-lasting improvement for ED.

Exosomes refer to a class of extracellular vesicles with a diameter of 50 to 100 nm, which are secreted by almost all cells.^17^ They usually encapsulate a complex payload containing lipids, signaling proteins, and nucleic acids, thus enabling cells to exchange information for multiple physiologic and pathologic functions.^18^ Accordingly, the beneficial effects of exosomes on ED in rat models have been found in recent experiments.^19^^,^^20^ Among these studies, exosomes are mostly derived from stem cells, including bone marrow–derived mesenchymal stem cells (BMSCs), adipose-derived mesenchymal stem cells (ADSCs), and human urine–derived stem cells. However, the value of stem cell–derived exosomes in ED treatment has not been comprehensively interpreted. We tried to explore whether exosomes derived from stem cells have therapeutic effects on ED in rat models. Additionally, we attempted to address the following problems: (1) Among exosomes derived from different stem cells—ADSCs, BMSCs, and human urine–derived stem cells, which have better therapeutic efficacy? (2) Among different ED models—diabetic mellitus, cavernous nerve injury, PD, artery injury, and chronic intermittent hypoxia, which can be ameliorated better by exosomes therapy?

## Methods

### Literature search strategy and selection criteria

This meta-analysis was conducted in accordance with the Preferred Reporting Items for Systematic Reviews and Meta-analyses.^21^ Literature retrieval was conducted in PubMed, Web of Science, and Embase for pertinent studies. We also utilized preprint databases, including bioRxiv and medRxiv, to find potential articles without peer review to avoid publication bias.

The keywords were as follows: (“stem cell” or “SC”) and (“exosomes” or “extracellular vesicles”) and (“erectile dysfunction” or “ED”). Additionally, we hand-searched the references of all relevant articles if necessary. We did not apply any language restrictions. Reviews, duplicates, conference abstracts, and clinical trials were excluded. Abstracts were screened for relevance, and the full texts were read when it was unclear from the abstracts.

The inclusion criteria were as follows: randomized/nonrandomized controlled animal experiment, rat/mouse model, and the utilization of exosomes to treat ED.

### Quality assessment

Two investigators were assigned to separately assess the methodological quality of included studies. The ARRIVE criteria^22^ and ESSM guidelines^23^ for reporting intracavernous pressure/mean artery pressure (ICP/MAP) were applied in assessment standards. There are 27 criteria, 1 point for each (not mentioned or unclear, 0; yes, 1). Studies with a score ≥18 were considered high quality, and studies with a score <18 were considered moderate quality.

### Data extraction

Data were extracted independently by 2 authors of our team. The third author was involved when the 2 independent authors disagreed and failed to reach consensus after referring to the original articles. The following information from each study was extracted: first author, year of publication, source of exosomes, exosome indicators, ED model, species, follow-up time, injection frequency, injection methods, dose of exosomes injected, and molecular changes after exosome therapy. ICP/MAP was the primary outcome. Structural changes were also collected: the ratio of smooth muscle to collagen (SM/collagen), CD31, alpha smooth muscle antibody (α-SMA), eNOS, neural nitric oxide synthase (nNOS), the apoptotic protein cleaved caspase 3, and transforming growth factor β1 (TGF-β1).

The mean and SEM or SD were extracted from the included article texts. The software Web Plot Digitizer (https://automeris.io/WebPlotDigitizer/) was used to extract numeric values from charts if final results were displayed only as graphs and we failed to receive a reply from the corresponding authors of articles.

### Statistical analyses

We used RevMan 5.3 software (The Nordic Cochrane Center) to analyze extracted data. To show the difference of ICP/MAP between the exosome therapy groups and the ED control groups, we used standardized mean difference with 95% CIs, which was also applied to structural changes in the corpus cavernosum, including SM/collagen and the expression of CD31, α-SMA, eNOS, nNOS, TGF-β1, and caspase 3 protein. Heterogeneity was evaluated with the *I*^2^ statistic test. A random effects model was adopted if *I*^2^ ≥ 50%, and a fixed effects model would be applicable if *I*^2^ < 50%. Stata (version 15.0; StataCorp) was used to examine publication bias with the Egger test^24^^,^^25^ and funnel plot. In addition, a *P* value <.05 in the Egger test was considered statistically significant for publication bias.

Meanwhile, subgroup analysis was used to investigate the possible source of heterogeneity among these studies. Subgroup analysis of ICP/MAP was based on 2 factors:

*Exosome source cell:* ADSCs vs BMSCs vs human urine–derived stem cells
*ED model type:* diabetic mellitus vs cavernous nerve injury vs PD vs artery injury vs chronic intermittent hypoxia resembling obstructive sleep apnea

## Results

### Study selection and characteristics

**Figure 1:**
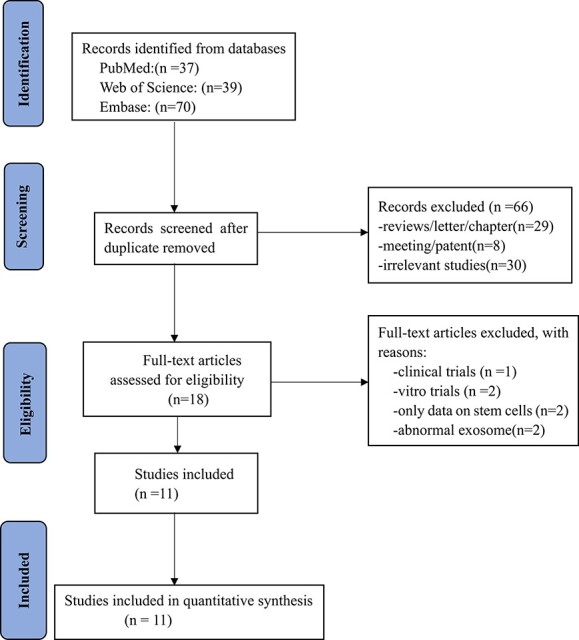
Flowchart of study selection.

As shown in Figure 1, 146 publications were identified after the search. We eventually enrolled 11 studies as a result of full-text review. The characteristics of eligible studies are described in Table 1.

**Table 1:** Characteristics of included studies.

**Year**	**First author**	**Producer cell**	**Isolation method**	**Exosome labels**	**Exosome dose, μg** ^**a**^	**ED model**	**Establishment method**	
2017	Chen^26^	ADSC	Multistep centrifugation	CD63, CD81, calnexin	100	T2DM	High-fat diet, intraperitoneal injection of low-dose STZ (30 mg/kg)	
2021	Liang^27^	ADSC	ExoQuick-TC reagent	CD9, CD63, TSG101	400	Hypoxia	Chronic intermittent hypoxia exposure	
2020	Song^32^	ADSC, BMSC	Multistep centrifugation	CD9, CD63, TSG101, calnexin	100	T1DM	Intraperitoneal injection of STZ (60 mg/kg)	
2018	Li^20^	BMSC, ADSC	PureExo exosome isolation kit	CD63, HSP70, CD81	100	CNI	Bilateral cavernous nerve crush injury	
2020	Wang^28^	ADSC	Ultracentrifugation, ultrafiltration	CD31, CD9, CD63, CD81	200	T1DM	Intraperitoneal injection of STZ (60 mg/kg)	
2019	Liu^31^	BMSC	Multistep centrifugation	CD9, TSG101	50 or 100	AI ^b^	Internal iliac artery ligation	
2018	Ouyang^19^	BMSC	Multistep centrifugation	CD63, TSG101, flotillin-1	100	CNI	Bilateral cavernous nerves crush injury	
2022	Liang^29^	ADSC	Differential centrifugation	CD63, CD9	150 ^c^	CNI	Bilateral cavernous nerves crush injury	
2018	Zhu^30^	ADSC	Exosome precipitation solution, ExoQuick	CD63, CD9	10 or 100	T1DM	Intraperitoneal injection of STZ (65 mg/kg)	
2020	Yang^33^	HUSC	Ultracentrifugation, ultrafiltration	CD9, CD63, TSG101, alix	100	PD	Intratunical injection of TGF-β1	
2019	Ouyang^34^	HUSC	Ultracentrifugation	CD63, calnexin	100	T1DM	Intraperitoneal injection of STZ (50 mg/kg)	
**Year**	**First author**	**Species**	**Animal age, wk**	**Injection method**	**Frequency**	**Follow-up, wk**	**Investigated parameters**	**Cargo**
2017	Chen^26^	SD rats	6	IC	1	4	ICP/MAP, CD31, Bcl-2, caspase 3	Not mentioned
2021	Liang^27^	SD rats	Not mentioned	IC	8	8	ICP/RT-AP, α-SMA, eNOS	miR-301a-3p
2020	Song^32^	SD rats	8	IC	1	4	ICP/MAP, NO, cGMP	Not mentioned
2018	Li^20^	SD rats	12	IC	1	3	nNOS, vWF,a-SMA, SM/collagen	Not mentioned
2020	Wang^28^	SD rats	8	Intravenous	1	2	ICP/MAP, ANP, BNP, nNOS	Corin
2019	Liu^31^	SD rats	12	IC	1	4	ICP/MAP, CD31, VEGFA, nNOS, eNOS, α-SMA, SM/collagen, collagen content	Not mentioned
2018	Ouyang^19^	SD rats	10	IC	1	4	ICP/MAP, nNOS, SM/collagen, caspase 3	Not mentioned
2022	Liang^29^	SD rats	6-8	IC	1	3	MICP/MAP, α-SMA, eNOS, nNOS	Not mentioned
2018	Zhu^30^	SD rats	10	Corpus cavernosum injection	1	4	ICP/MAP, SM/collagen, endothelial content	microRNAs
2020	Yang^33^	SD rats	Not mentioned	Intratunical	1	4	ICP/MAP, elastin, TGF-β1, collagen III, SM/collagen, Smad2/3 protein	Not mentioned
2019	Ouyang^34^	SD rats	Not mentioned	IC	1	4	CD31, eNOS, phospho-eNOS, nNOS, SM/collagen	miRNA

Abbreviations: α-SMA, alpha smooth muscle actin; ADSC, adipose-derived mesenchymal stem cell; ANP, pro-atrial natriuretic peptide; BMSC, bone marrow–derived mesenchymal stem cell; BNP, pro-brain natriuretic peptide; CD31, cluster of differentiation 31; cGMP, cyclic guanosine monophosphate; CNI, cavernous nerve injury; ED, erectile dysfunction; eNOS, endothelial nitric oxide synthase; HUSC, human urine–derived stem cells; IC, intracavernous injection; ICP/MAP, intracavernous pressure/mean artery pressure; MICP, maximum intracavernous pressure; nNOS, neuronal nitric oxide synthase; NO, nitric oxide; PD, Peyronie disease; RT-AP, real-time carotid arterial pressure; SD, Sprague-Dawley; SM/collagen, ratio of smooth muscle to collagen; STZ, streptozotocin; T1DM, type 1 diabetes mellitus; T2DM, type 2 diabetes mellitus; TGF-β1, transforming growth factor β1; vWF, von willebrand factor.

^a^The amount of stem cell–derived exosomes dissolved in phosphate-buffered saline.

^b^Artery injury by bilateral internal iliac artery ligation.

^c^This study utilized a polydopamine nanoparticle–incorporated poly(ethylene glycol)-poly(ε-caprolactone-co-lactide) gel as a solvent of exosomes.

Exosomes derived from ADSCs were applied in 5 studies,^26–30^ and exosomes derived from BMSCs were used in 2 studies.^19^^,^^31^ Both parental cells were utilized in another 2 studies.^20^^,^^32^ The remaining 2 studies exploited exosomes derived from human urine–derived stem cells.^33^^,^^34^

Regarding ED models, 5 studies^26^^,^^28^^,^^30^^,^^32^^,^^34^ injected streptozotocin into animals to establish diabetic mellitus model, in which 1 study constructed a type 2 diabetic mellitus model by high-fat diet. Three studies^19^^,^^20^^,^^29^ constructed a neurogenic ED model by damaging cavernous nerves surgically. Injection of TGF-β1 into rat tunica albuginea was utilized in 1 study to create a PD model,^33^ and 1 research utilized chronic intermittent hypoxia to mimic obstructive sleep apnea–induced ED.^27^ Another study focused on artery injury–indued ED.^31^

Administration of exosomes by intracavernous injection was used in 9 studies, while the other 2 studies used intratunical or intravenous injection.

### Quality assessment

The quality score of 6 studies was ≥18, and the other 5 studies received <18 points. Details are shown in Table 2.

**Table 2:** Quality assessment of included studies.

**Year**	**Study**	**Study design**		**Sample Size**		**Inclusion and exclusion**			**Randomization**				**Statistic**			
		**Control**	**Experiment unit**	**No.**	**Calculation**	**Criteria**	**Exclusion**	**No.**	**Randomization**	**Confounder**	**Blinding**	**Outcome measure**	**Methods, software**	**Assumption**		
2017	Chen^26^	1	1	1	0	1	0	1	1	0	0	1	1	0		
2021	Liang^27^	1	1	1	0	1	0	1	1	0	0	1	1	0		
2020	Song^32^	1	1	1	0	1	1	1	0	0	0	1	1	0		
2018	Li^20^	1	1	1	0	1	0	1	1	0	0	1	1	0		
2020	Wang^28^	0	1	0	0	1	0	0	0	0	0	1	1	0		
2019	Liu^31^	1	1	1	0	1	0	1	1	0	0	1	1	0		
2018	Ouyang^19^	1	1	1	0	1	0	1	0	0	0	1	1	0		
2022	Liang^29^	1	1	0	0	0	0	0	1	0	0	1	0	0		
2018	Zhu^30^	1	1	1	0	1	0	0	1	0	0	1	1	0		
2020	Yang^33^	1	1	1	0	1	0	1	1	0	0	1	1	0		
2019	Ouyang^34^	1	1	1	0	1	1	1	1	0	0	1	1	0		
**Year**	**Study**	**Animals**		**Procedures**				**Results**	**ICP/MAP**			**Cell**	**Exosome**			
		**Species**	**Further**	**What**	**When**	**Where**	**Why**	**Summary**	**Nommalization by MAP**	**Images of traces**	**APO test** ^**a**^	**Phenotype** ^**b**^	**Label**	**Morphology**	**Structural changes**	**Score**
2017	Chen^26^	1	1	1	1	1	1	1	1	1	0	0	1	1	1	20
2021	Liang^27^	1	0	1	1	1	1	1	1	1	0	1	1	1	1	20
2020	Song^32^	1	0	1	1	0	1	1	1	1	1	1	1	1	1	20
2018	Li^20^	1	0	1	1	0	1	1	1	1	0	0	1	1	1	18
2020	Wang^28^	1	0	1	1	0	1	1	1	1	0	1	1	1	1	15
2019	Liu^31^	1	0	1	1	0	1	1	1	1	0	0	1	1	1	18
2018	Ouyang^19^	1	0	1	1	0	1	1	1	1	0	1	1	1	1	18
2021	Liang^29^	1	0	1	0	0	1	1	1	1	0	0	1	1	1	13
2018	Zhu^30^	1	0	1	1	0	1	1	1	1	0	0	1	1	1	17
2020	Yang^33^	1	0	1	1	0	1	1	1	1	0	1	1	1	1	19
2019	Ouyang^34^	1	0	1	1	1	1	1	1	1	1	0	1	1	1	21

Abbreviation: ICP/MAP, intracavernous pressure/mean artery pressure.

^a^Apomorphine test to verify erectile dysfunction before administration of exosomes.

^b^Stem cell phenotype identification.

## Meta-analysis

### Intracavernous pressure/mean artery pressure

**Figure 2:**
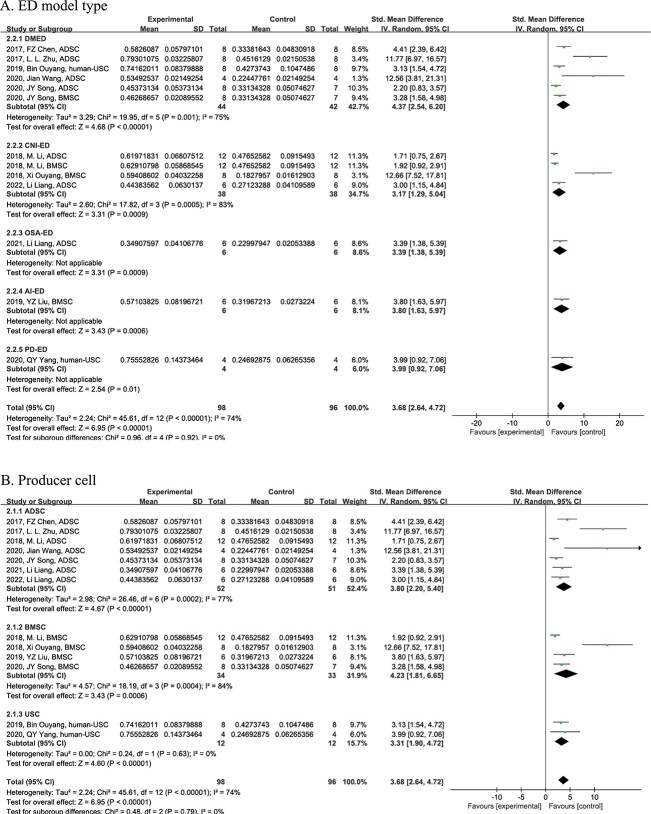
Forest plot for the ICP/MAP changes among different subgroups: (A) ED model type and (B) producer cell. ED, erectile dysfunction; ICP/MAP, intracavernous pressure/mean artery pressure.

The pooled analysis showed that stem cell–derived exosome therapy ameliorated ICP/MAP significantly (n = 194; standardized mean difference, 3.68; 95% CI, 2.64-4.72; *Z* = 6.95, *P* < .01; χ^2^ = 45.61, *I*^2^ = 74%), which hints at the improvement of erectile function (Figure 2A).

Subgroup analysis of ICP/MAP was conducted on the basis of 2 factors: ED model and producer cell. First, the analysis showed that in different ED model types, an increase of ICP/MAP occurred after administration of exosomes as compared with respective controls (diabetic mellitus, *P* < .01; cavernous nerve injury, *P* < .01; obstructive sleep apnea, *P* < .01; artery injury, *P* < .01; PD, *P* < .01). However, it showed no statistically significant difference in growth among different ED models (χ^2^ = 0.96, *P* = .92) (Figure 2A). Second, exosomes generated by different stem cells could all enhance ICP/MAP vs various controls (ADSCs, *P* < .01; BMSCs, *P* < .01; human urine, *P* < .01). However, no statistically significant difference was observed in producer cell types (χ^2^ = 0.48, *P* = .79) (Figure 2B).

### Structural changes

To investigate the underlying mechanism of exosome therapy for ED, structural changes were analyzed. Stem cell–derived exosomes restored SM/collagen (n = 144, *P* < .01), CD31 (n = 44, *P* < .001), α-SMA (n = 82, *P* < .006), nNOS (n = 82, *P* = .03), and eNOS (n = 58, *P* < .001) damaged by ED, which indicated the amelioration of endothelium and smooth muscle content of cavernosum. Furthermore, the decreases of TGF-β1 (n = 38, *P* = .003) and caspase 3 (n = 32, *P* < .001) were observed in the analysis, which meant that exosomes might improve cavernosum structures by inhibiting fibrosis and apoptosis (Table 3).

**Table 3:** Analyses of structural changes.

**Biomarker**	**No.**	**SMD**	**95% CI**	***Z***	***P* value**	**χ** ^**2**^	***I*** ^**2**^ **,%**
SM/collagen	144	3.71	3.10, 4.32	11.92	<.001	12.91	38
CD31	44	5.32	3.86, 6.78	7.14	<.001	2.73	27
α-SMA	82	3.57	1.01, 6.14	2.73	.006	36.3	86
eNOS	58	3.27	2.39, 4.15	7.32	<.001	1.6	0
nNOS	82	2.12	0.22, 4.03	2.19	.03	41.75	88
TGF-β1	38	−4.3	−7.17, −1.43	2.94	.003	8.48	76
caspase 3	32	−4.42	−5.86, −2.99	6.04	<.001	0.56	0

Abbreviations: α-SMA, alpha smooth muscle actin; CD31, cluster of differentiation 31; eNOS, endothelial nitric oxide synthase; nNOS, neuronal nitric oxide synthase; SM/collagen, ratio of smooth muscle to collagen; SMD, standardized mean difference; TGF-β1, transforming growth factor β1.

### Bias assessment

**Figure 3:**
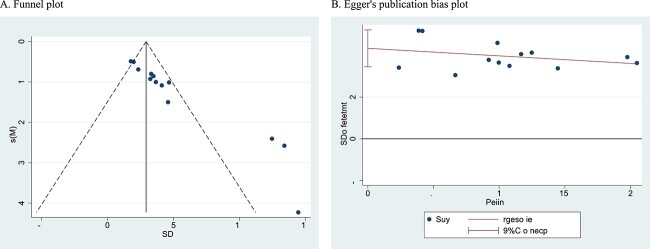
Publication bias test of ICP/MAP: (A) funnel plot and (B) Egger publication bias plot. ICP/MAP, intracavernous pressure/mean artery pressure.

The funnel plot appeared to be asymmetrical, which indicated that there was publication bias in the ICP/MAP analysis. Furthermore, the Egger test was used to detect publication bias, and its *P* value (*t* = 10.77, *P* < .05) showed bias from small study effects (Figure 3**)**.

## Discussion

A total of 11 published preclinical studies were included in our analysis. Overall, our analysis suggests that stem cell–derived exosomes could ameliorate ED and structural changes in various types of ED models.

Penile erection is a series of vascular events closely related to the endothelium and smooth muscle cells of the corpus cavernosum, which histologically form the basic structure of sinusoids. When the smooth muscle is contracted, the blood inflows through the cavernous artery restrictively, but it outflows through the subtunical venular plexus freely, resulting in a flaccid state of the penis.^35^ Upon sexual stimulation, nonadrenergic noncholinergic nerve fibers release nitric oxide (NO), which activates guanylyl cyclase to increase the concentration of cGMP (cyclic guanosine monophosphate). Furthermore, acetylcholine released from parasympathetic cholinergic nerve fibers causes activation of adenylyl cyclase, increasing the concentration of cAMP (cyclic adenosine monophosphate). High levels of cGMP and cAMP decrease intracellular Ca2+ levels and lead to smooth muscle cell relaxation, followed by a normal erection. If any of these processes are interrupted, ED may happen. For example, cavernous nerve injury causes downregulation in the nerve signaling of the corpora cavernosa, which reduces the NO level in smooth muscle, increases apoptosis in the smooth muscle and endothelium of blood vessels, and upregulates fibrogenetic cytokines to form collagenization of the smooth muscle. These functional and structural changes lead to veno-occlusive dysfunction.^36–38^ Hypoxia can cause a decrease in prostaglandin E1 levels of the corpora cavernosa, which commonly inhibits profibrotic cytokines such as TGF-β1.^39^^,^^40^ These profibrotic cytokines enhance collagen deposition, decrease the smooth muscle content, reduce the elasticity of the penis, and impair the ability of the cavernosa to compress the subtunical veins, causing veno-occlusive dysfunction.^36^ As reported, the mechanisms of diabetic ED observed in rat models may include elevated glycation end products and oxygen-free radical levels, which impaired synthesis of nNOS and decreased cGMP-dependent kinase 1.^41^^,^^42^ In a word, ED is a multifactorial condition with a complex neurovascular process, which is strongly associated with the loss and dysfunction of the corporal endothelium and smooth muscle.

Clinically, refractory male ED shows resistance to drug therapy. Facing this obstacle, stem cell therapy is recognized as a promising novel method in ED treatment, and considerable studies have proved its feasibility in animal models and clinical trials,^43^^,^^44^ which may be attributed to their capability of self-renewal, proliferation, and multipotential differentiation. Moreover, the regenerative properties have been established in tissue engineering and regenerative medicine research.^45^^,^^46^

Some studies recently considered that the beneficial effects of transplanted stem cells could not be merely explained by engraftment or differentiation into specific cells.^47^ Scientists have paid more attention to the paracrine secretion of stem cells, including chemoattractant molecules, bioactive factors, and extracellular vesicles.^48^^,^^49^ Exosomes are 50- to 100-nm membrane-bound extracellular vesicles, in which content varies depending on the original cells and the activation status, including noncoding small RNAs, mRNAs, proteins, and lipids.^50^ Exosomes have been proved to serve multiple physiologic and pathologic functions via regulating intercellular communication.^51^ Lai et al^52^ reported that exosomes derived from mesenchymal stem cells (MSCs) exerted a protective effect on cardiac tissue following myocardial infarction. Zhang et al^53^ demonstrated that MSC-derived exosomes effectively promoted functional recovery in rats after traumatic brain injury by facilitating endogenous angiogenesis and neurogenesis. When compared with stem cell therapy, exosomes have many advantages, such as greater stability and ease of storage and management, preclusion of the risk of tumor formation, and a lower likelihood of an immune rejection.^19^^,^^26^

Similar to stem cell therapy for ED, our analysis showed that stem cell–derived exosomes increased SM/collagen and the expression of α-SMA, CD31, nNOS, and eNOS damaged in ED. CD31 can be considered a biomarker of endothelium contents,^54^ while α-SMA and SM/collagen indicated the smooth muscle contents in the corpus cavernosum of rats. This hints that exosomes could improve the tissue structure of the corpus cavernosum to ameliorate erectile function. Moreover, our study suggested the downregulated expression level of TGF-β1 and caspase 3. As a kind of profibrotic cytokine, TGF-β1 was recognized as a key factor related to the formation and development of corporal fibrosis as in PD.^55^ Kim et al reported that the activation of TGF-β1 signaling initiated collagen accumulation and deposition.^56^ The antifibrotic effect of exosomes has been demonstrated by some studies in different diseases, such as liver and lung fibrosis.^57^^,^^58^ The downregulation of TGF-β1 in our analysis may hint that the exosomes can also have an antifibrotic effect on ED. Activation of caspases was recognized as the biochemical marker for apoptosis, which is widely used in apoptotic signals examination.^59^ Vasculogenic ED induced by artery injury was characterized as the ischemic and hypoxic state of the corpus cavernosum, which may increase the release of reactive oxygen species, leading to cell apoptosis.^60^^,^^61^ It is reported that oxidative stress in penile ischemia is an important factor in ED progress.^60^ In our analysis, the administration of stem cell–derived exosomes decreased the expression level of caspase 3 and TGF-β1, which indicated that exosomes possess the ability to inhibit fibrosis and apoptosis, ensuring the functional endothelium and smooth muscle contents in the corpus cavernosum. The NO/cGMP signaling pathway was important to regulate penile erection, and downregulation of this pathway contributed to ED.^62^ NO produced by eNOS in cavernous endothelial cells and nNOS in cavernous nerves induce erection by increasing the cGMP content in the smooth muscle cells of the corpus cavernosum.^35^^,^^63^ Song et al reported that exosomes derived from smooth muscle cells regulated the NO/cGMP pathway to ameliorate ED.^32^ The levels of eNOS and nNOS significantly increased after exosome therapy in our analysis, which indicated that stem cell–derived exosomes might make functional changes in the corpus cavernosum via the NO/cGMP signaling pathway.^32^ The outcome was consistent with the change of ICP/MAP.

In our meta-analysis, only 2 studies used exosomes generated from human urine–derived stem cells^33^^,^^34^; the others used exosomes derived from ADSCs or BMSCs. In our analysis, 3 kinds of exosomes showed no difference in the therapeutic efficacy. Although exosomes can be generated by most cells, the exosomes derived from MSCs were used in most research to treat ED. MSCs can be isolated from several tissues, such as bone marrow, adipose tissue, Wharton jelly tissue, umbilical cord blood, and neonatal teeth.^64^ Among them, adipose-derived stem cells and bone marrow–derived stem cells were exploited the most. Noncoding RNAs, such as miRNA, snoRNA, and tRNA, enriched in the exosomes produced by stem cells, may exert important biological functions by conveying properties of parental cells. For example, tRNAs accounted for >50% of the total small RNAs in the exosomes derived from ADSCs, as opposed to merely 23% to 25% in BMSC-derived exosomes.^65^ Interestingly, some specific tRNAs were more abundant in exosomes than the source cells. It has been proved that miRNAs were the major content of cellular small RNA in MSCs, and the discrepancy might suggest preferential sorting and release.^50^^,^^65^ Moreover, even exosomes originating from the same parental cells might exhibit heterogeneity of content.^66–68^ The subcellular origin and cell activation status were responsible for the molecular heterogeneity of exosomes.^69^^,^^70^ Due to the limitation of exosome isolation methods, bulk isolates rather than pure exosome population isolates were used in a majority of studies upon evaluation of their therapeutic efficacy.^50^ Exosomes isolated from urine also contained substantial noncoding small RNAs, such as tRNA and rRNA, while the exact functions need further study. The research on exosome-mediated communication mostly focused on well-known RNA species, such as miRNAs and mRNAs, for the sake of detection sensitivity and specificity of exosome contents.^50^ Zhu et al^30^ found that ADSC-derived exosomes contained some microRNAs with proangiogenic (miR-126, miR-130a, and miR-132) and antifibrotic (miR-let7b and miR-let7c) functions. Simultaneously, proteins on the membrane of and in the vesicles get involved in inter- and intracellular signaling mediation. Wang et al^28^ used the transmembrane serine protease corin in ADSC-derived exosomes to improve ED in diabetic rats and suggested that it may play a role through the ANP/NO/cGMP signaling pathway.

To the best of our knowledge, this is the first meta-analysis providing comprehensive insights into the effects of stem cell–derived exosomes on ED in rats. The value of a systematic review of experimental animal studies has been steadily understood.^71^^,^^72^ The consistent results of exosome therapy efficacy across various ED models in our study could provide reassurance that human beings might also respond in the same way.

There are still several limitations in this study. A high degree of heterogeneity remains in ICP/MAP outcome after subgroup analysis. This may be attributed to the methodological heterogeneity of the studies. Specifically, the exosome types, extraction methods, and animal models used in studies were quite different. Given the limited number of included studies, a persuasive subgroup analysis cannot be performed. Moreover, different software (eg, SPSS, GraphPad Prism, and Stata) was applied across the studies, which may cause high statistical heterogeneity.

The Egger test shows publication bias in the analysis, which may be attributed to the fact that articles with negative conclusions are less likely to be published and retrieved. However, we tried our best to retrieve animal intervention studies on exosome treatment for ED, including preprint databases such as bioRxiv and medRxiv, but failed to find more relevant research, perhaps because exosome therapy for ED is a relatively new topic. Despite the existence of publication bias, our analysis still shows the therapeutic effects of exosomes on ED.

## Conclusion

This meta-analysis reveals the therapeutic effects of stem cell–derived exosomes on ED rat models. Exosome administration may improve erectile function by activating the NO/cGMP signaling pathway, ameliorating endothelium, and inhibiting the fibrosis and apoptosis of cavernosum. Stem cell–derived exosomes have great potential to afford a novel cell-free therapy for ED. However, further studies are needed to identify the functional components of exosomes, and clinical trials may be worthwhile to demonstrate the actual effects on the human body.

## Funding

This work was supported by the National Natural Science Foundation of China (No. 81871157).

*Conflicts of interest:* The authors report no conflicts of interest.
